# Pilomatrixoma in the cheek: a clinical case report

**DOI:** 10.4322/acr.2021.387

**Published:** 2022-06-23

**Authors:** Mario Vitor Carcassola, Wladimir Gushiken de Campos, Celso Augusto Lemos, Basilio de Almeida Milani, Ricardo Luiz Psciolaro, Marcelo Minharro Ceccheti

**Affiliations:** 1 Hospital Doutor Fernando Mauro Pires da Rocha - Hospital do Campo Limpo, Department of Oral and Maxillofacial Surgery, São Paulo, SP, Brasil; 2 Universidade de São Paulo (USP), School of Dentistry, Department of Stomatology, São Paulo, SP, Brasil

**Keywords:** Pilomatrixoma, Skin neoplasms, Neoplasms, Pathology, Oral surgery

## Abstract

Pilomatrixoma is a follicular benign tumor of unknown etiology that originates in the matrix of a hair follicle. It develops more frequently in children and young adults, with a slight predominance in female patients. It is a slow-growing tumor that presents as a mobile nodule, firm, and with well-defined borders. The present study aimed to report the clinical, histopathologic, and radiographic aspects of pilomatrixoma in the cheek area of a 20-year-old female patient as the established treatment at an oral maxillofacial department. Pilomatrixoma is rarely included in the differential diagnosis of masses and nodules in the skin, and it is often confused with other nodular lesions, such as epidermoid cysts. The diagnostic method to identify this entity is an incisional biopsy. Because of its high incidence in the head and neck region, oral surgeons should be well-acquainted with this type of tumor so that it can be included as a diagnostic hypothesis of masses and nodules of the head and neck.

## INTRODUCTION

The etiology of pilomatrixoma, or pilomatricoma, has not yet been clarified, but evidence suggests a mutation in the *CTNNB1* gene, which encodes the beta-catenin protein. It is a benign follicular cutaneous neoplasia originating in the cells of the hair follicle matrix.^1^^,^^2^

The lesion was first described by Malherbe^3^ in 1880, with the name “calcifying epithelioma.” About eighty years later, Forbis and Helwig^4^ renamed it pilomatrixoma as the term “epithelioma” usually referred to as a malignant lesion.

The incidence of dermatological lesions is still uncertain and scarcely studied, with reports of indices ranging from 0.001% to 0.12% of all studied dermatological material.^1^^,^^5^ It is more common in children and young adults and is more prevalent in the first decade of life. The most frequent location is the head and neck region. Pilomatrixoma usually present as isolated lesions and are clinically described as a hardened, well-defined, mobile lesion located in the subcutaneous tissue.^6^ It is a lesion frequently confused with other skin entities and is rarely included among the differential diagnoses of masses and skin nodules.^1^

Surgical excision is the treatment described in the literature. There are few reported cases of surgical curettage, but without long-term follow-up. This procedure has an excellent prognosis with low recurrence rates (between 0% and 2.63%).^1^^,^^4^^,^^6^

This study aims to report a case of pilomatrixoma located in the maxillofacial region and to discuss its clinical, radiological, histopathological, and surgical features.

## CASE REPORT

A 20-year-old white female patient complained of a progressive bulging of the right jugal region over the last seven months. She reported that 4 months after noticing the lesion, she had an episode of edema and pain in the affected region, which was controlled with analgesics and non-steroidal anti-inflammatory drugs.

**Figure 1:**
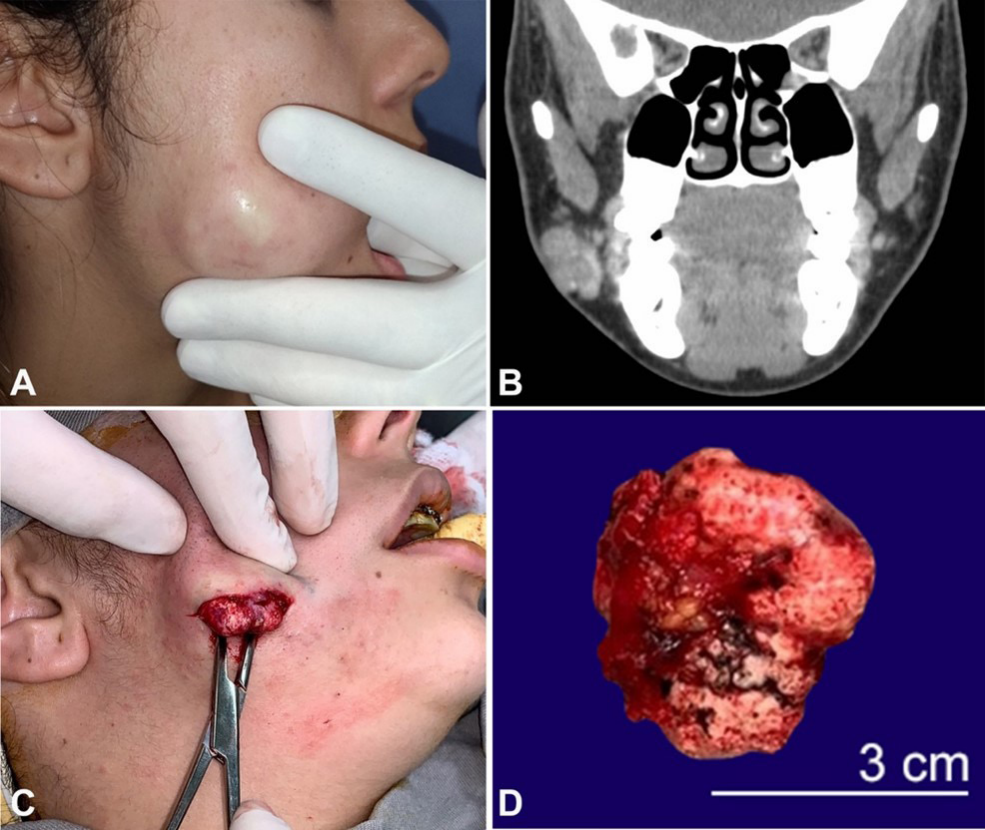
**A** – Clinical aspect of the lesion. Details for regions of discoloration (ischemia) of the skin when the lesion is delimited and compressed against the buccal mucosa; **B** – contrasted CT examination showing the presence of a nodule with lobulated contours and tiny calcifications of subcutaneous permeation, reaching a dimension of 2.6 cm; **C** – dissection was performed using delicate Kelly forceps to separate the lesion capsule from adjacent muscle tissues and skin for total lesion excision in a single fragment; **D** – Macroscopic aspect of the lesion. A solid, well-defined lesion measuring approximately 24 mm × 14 mm × 12 mm.

On physical examination, she presented with a well-defined nodule in the right cheek region, approximately 2.5 cm in the largest diameter, hardened, painless on palpation, and not fixed to the deep tissues. The lesion was superficial and the overlying skin became ischemic when finger-pressed (Figure 1A).

Intravenous contrast-enhanced CT showed a mixed image with the presence of hyperdense dots attached to the adjacent skin (Figure 1B). Clinical and imaging data determined the diagnostic hypothesis of a dermoid or epidermoid cyst.

The patient underwent incisional biopsy under local anesthesia by cutaneous access.

**Figure 2:**
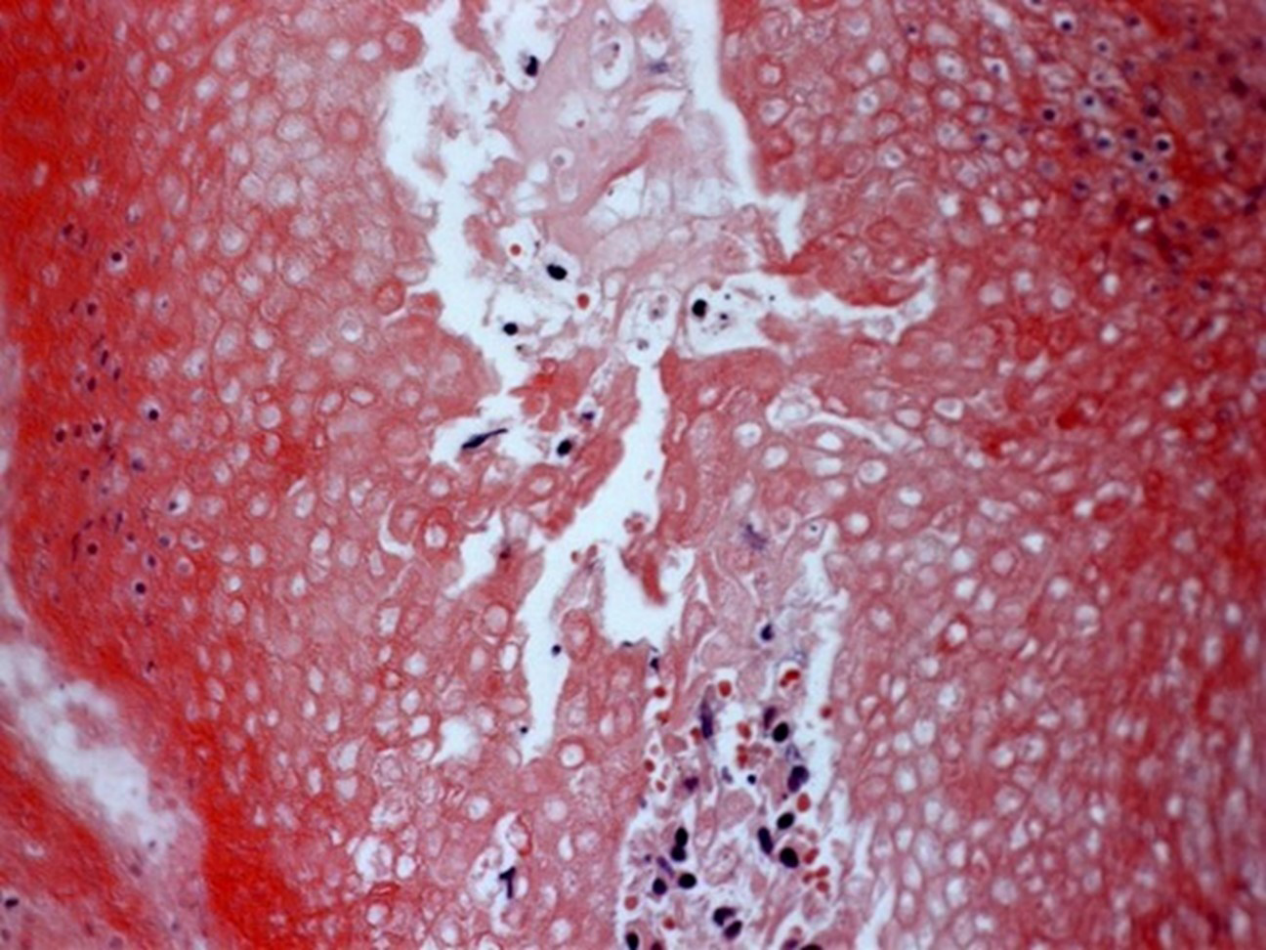
Photomicrograph of the tumor showing the presence of multiple cells without a nucleus (ghost cells) (H&E, 400×).

The treatment proposed was a complete surgical excision. Under general anesthesia, an extraoral incision of approximately 3 cm in the caudal part of the nodule was performed. The lesion had dimensions of approximately 24 mm × 14 mm × 12 mm (Figure1C and 1D). Histological sections revealed a fragment of cutaneous neoplasia characterized by nests of epithelium with basaloid cells that underwent an abrupt transition to a completely keratinized epithelium, rich in ghost cells, with confirmation of pilomatrixoma (Figure 2). No signs of malignancy were observed.

## DISCUSSION

In 1973, Moehlenbeck^5^ conducted a statistical survey of 1,569 cases of pilomatrixoma and found that more than 40% of the tumors appeared before the age of 10 years and the remainder before the third decade of life, with a higher incidence between the ages of 8 and 13 years. Women were more affected than men, and the ratio of occurrence was 3:2. However, in a recent literature review,^1^ the predominance in women was milder than in men (1.15:1). In the present case, the patient was a woman. Her age (20 years) was above the age group most affected, perhaps due to late diagnosis.

The most frequent lesion location is the head, with an incidence between 39.6% and 51.5%, followed by the neck, upper extremities, trunk, and lower extremities.^2^^,^^4^^-^6 On the face, the most affected region is the cheek accounting from 18.8% to 36% of all cases, followed by the periorbital region with an incidence between 6.6% and 14%.^2^^,^^7^

The lesions are typically solitary, with multiple manifestations occurring in 1.9% to 8.2% of cases.^2^^,^^5^^-^7 Multiple lesions may be associated with genetic diseases, such as myotonic dystrophy.^1^^,^^2^ Clinically, the lesion presents itself as a slow-growing, hardened, subcutaneous, mobile nodule, with regions of discoloration of the skin that can be evidenced through finger pressure. In 35% of patients, there were painful symptoms^2^ apart from the episode of pain mentioned by the patient in this study, there was no pain on palpation and manipulation of the lesion. In some cases, ulceration and/or perforation of the skin may occur. There are several clinical variations, such as basal cell carcinoma and squamous cell carcinoma, which can be considered as a differential diagnosis of pilomatrixoma.^6^ Lesions typically range from 0.4 mm to 60 mm.^1^^,^^2^^,^^6^ In the present case, the lesion was solitary and had measured 24 mm. The clinical aspect of the reported lesion is the most common subtype found in the literature, with no ulcerations and/or features that could lead to a differential diagnosis of a malignancy.

On the histological evaluation of pilomatrixoma, the matrix cells present a slightly basophilic cytoplasm with a large, oval nucleus and a prominent nucleolus. A transition from these cells in the matrix to ghost cells is observed. Other components include multinucleated giant cells, nucleated squamous cells, and chronic inflammatory infiltrate.^8^ The presence of basaloid and ghost cells, which are characterized as cells without a nucleus, appears to be a key component in the diagnosis of the lesion.^8^ In the histopathological examination of the reported case, transition from basaloid cells to ghost cells and the presence of multinucleated giant cells were observed, which were highly suggestive of pilomatrixoma.

The most commonly used complementary imaging modality is an ultrasound, with a diagnostic rate of 15.3%,^9^ followed by CT with intravenous contrast^10^, which shows a well-defined subcutaneous mass with calcifications,^1^ the latter was used in the present study, to the diagnosis and surgical planning. Moreover, radiographs have little diagnostic value for pilomatrixomas.

The definitive treatment of pilomatrixoma is total surgical excision. When the lesion is tightly adherent to the adjacent skin, it is necessary to remove the skin together with the lesion. In the present case, the lesion was easily dissected from the skin.

## CONCLUSIONS

Pilomatrixoma is an uncommon lesion that is easily confused with other pathologies. Oral surgeons must be able to recognize and differentiate this lesion to include it as a diagnostic hypothesis in head and neck lesions.

## References

[1] Jones CD, Ho W, Robertson BF, Gunn E, Morley S (2018). Pilomatrixoma: a comprehensive review of the literature. Am J Dermatopathol.

[2] Pirouzmanesh A, Reinisch JF, Gonzalez-Gomez I, Smith EM, Meara JG (2003). Pilomatrixoma: a review of 346 cases. Plast Reconstr Surg.

[3] Malherbe A, Chenantais J (1880). Note surl’épitheliomacalcifié des glandes sébacés. Progrèsmédical (Paris).

[4] Forbis R, Helwig EB (1961). Pilomatrixoma (Calcifying Epithelioma). Arch Dermatol.

[5] Moehlenbeck FW (1973). Pilomatrixoma (calcifying epithelioma). A statistical study. Arch Dermatol.

[6] Julian CG, Bowers PW (1998). A clinical review of 209 pilomatricomas. J Am Acad Dermatol.

[7] Agarwal RP, Handler SD, Matthews MR, Carpentieri D (2001). Pilomatrixoma of the head and neck in children. Otolaryngol Head Neck Surg.

[8] Wang J, Cobb CJ, Martin SE, Venegas R, Wu N, Greaves TS (2002). Pilomatrixoma: clinicopathologic study of 51 cases with emphasis on cytologic features. Diagn Cytopathol.

[9] Lin SF, Xu SH, Xie ZL (2018). Calcifying epithelioma of malherbe (pilomatrixoma): clinical and sonographic features. J Clin Ultrasound.

[10] Jones C, Twoon M, Ho W, Portelli M, Robertson BF, Anderson W (2018). Pilomatrix Carcinoma: 12-year experience and review of the literature. J Cutan Pathol.

